# Urinary phoretograms performed by capillary electrophoresis in dogs with chronic disease with or without *Leishmania infantum* infection

**DOI:** 10.3389/fvets.2022.979669

**Published:** 2022-11-18

**Authors:** Paula Fátima Navarro, Salceda Fernández-Barredo, Laura Gil

**Affiliations:** ^1^Department of Veterinary Medicine and Surgery, Universidad Católica de Valencia San Vicente Mártir, Valencia, Spain; ^2^CEDIVET (Centro Diagnóstico Veterinario), Valencia, Spain

**Keywords:** *Leishmania* infantum, urinary phoretogram, canine leishmaniasis, proteinuria, chronic kidney disease, renal, urinary, capillary electrophoresis

## Abstract

**Introduction:**

The study of early markers to detect kidney malfunction has increased in recent years since serum markers, such as creatinine increase when there is a 75% loss of renal mass. Urinary capillary electrophoresis (UCE) is an available laboratory technique that provides an easily interpretable electrophoretic pattern. This pattern in our study has been divided into five fractions as it is done in serum: fraction 1 migrating in the albumin zone, fraction 2 in the alpha_1_-globulins zone, fraction 3 in the alpha_2_-globulins zone, fraction 4 in the beta-globulins zone, and fraction 5 in the gamma globulins zone. UCE can be useful in the early diagnosis of renal disease.

**Material and methods:**

In this study, UCE was performed in dogs with azotemia and proteinuria due to chronic kidney disease (CKD) not related to *Leishmania infantum* (*L. infantum*) infection (G_1_, *n* = 11) and dogs with CKD related to *L. infantum* infection (G_2_, *n* = 17) and compared with reference intervals from healthy dogs (G_0_, *n* = 123), with the aim of comparing their phoretograms and assessing changes in the fractions of the phoretograms based on the health status of individuals.

**Results:**

Fraction 2 was statistically augmented in dogs with CKD (G_1_) when compared with the healthy population (G_0_) and dogs infected by *L. infantum* (G_2_). Fraction 3 was statistically increased in dogs with CKD (G_1_) and dogs infected by *L. infantum* (G_2_) compared with G_0._ Fraction 4 was found to be statistically decreased in dogs with CKD (G_1_) and dogs infected by *L. infantum* (G_2_) compared with G_0._ Fraction 5 was statistically higher in dogs with *L. infantum* (G_2_) compared with G_0_ and dogs with CKD (G_1_). No statistical relationship was found between the protein to creatinine ratio and different fractions from the urinary phoretogram in the study population. No statistical relationship was found between serum and urine fractions in the study population.

**Discussion:**

The results of the present study suggest that UCE is a promising non-invasive technique that might be used as a part of the diagnostic and follow-up in dogs with kidney disease due to different pathologies.

## Introduction

The study of urinary biomarkers in the early detection of kidney disease has increased considerably in the last few years (1–7). Evaluation of protein loss in urine can be carried out by semiquantitative methods, such as urinary reactive strips or quantitative methods such as the measurement of albumin or microalbumin concentration and the protein to creatinine (UPC) ratio in urine (8). Proteinuria in dogs is considered significant by the IRIS guidelines when UPC is >0.5 accompanied by inactive sediment (9).

Electrophoresis is a laboratory technique based on the separation of biomolecules according to their charge, molecular weight, and structure. The biomolecules are subjected to an electric field, thus causing a migration of the cathode or the anode. Over time, the most widely used electrophoresis was agarose gel electrophoresis, but this has gradually been replaced by capillary electrophoresis (10, 11).

Capillary electrophoresis (CE) is based on the separation of molecules according to the speed of migration, under the action of an electric field with ionic compounds. The separation is performed through a small diameter fused silica capillary (12, 13). The advantages of capillary electrophoresis over other laboratory electrophoretic techniques are easily reproducible and is a fast and highly sensitive technique that does not require additional stains. The disadvantages are the high economic cost of the equipment and the use of voltages that can lead to abnormal results (10, 14). This laboratory technique has been used in human medicine for the detection of metabolic diseases (15). It has also been used in the detection of inflammatory, infectious, immune-mediated, and neoplastic diseases in both human and veterinary medicine (16, 17). In veterinary medicine, capillary electrophoresis is a field still under study. Different studies have been carried out on the use of this analytical technique in serum and urine, but agarose gel electrophoresis is still the most widely used technique for the study of proteinuria in kidney diseases.

Canine leishmaniosis is a zoonotic pathology that can affect dogs among other species. The clinicopathological alterations of canine leishmaniosis include the development of non-regenerative normochromic normocytic anemia, thrombocytopenia, or changes in the leukogram. Serum biochemistry and urinalysis may indicate renal dysfunction (such as azotemia, decreased urine specific gravity, and proteinuria) as well as an inflammatory/immune response (increased acute phase proteins or alpha_2_-globulins and/or gamma globulins). Dogs with active leishmaniosis generally have high antibody titers, whereas low titers predominate in infected and immunologically resistant dogs or exposed dogs without confirmation of parasite presence. Quantitative serology is recommended for clinically suspicious dogs, as high antibody titers can confirm the clinical diagnosis. In confirmed and treated dogs, renal function and inflammatory/immune response variables should be monitored periodically (18). In dogs with leishmaniosis and proteinuria, several studies have been carried out by gel electrophoresis with the aim of describing the different biomarkers contained in the urine (6, 18, 19). In one study, IgG and IgA were found in dogs with *L. infantum* confirming both glomerular and tubular damage (6), and another study confirmed the presence of a high GGT/urinary creatinine ratio indicating tubular damage (19). There are only two studies about the use of UCE in dogs. One of these studies evaluates urinary peptides excreted for the diagnosis of chronic kidney disease, while the other study establishes reference intervals for protein fractions in the urine of healthy animals in order to make a comparison with pathological processes (16, 20).

The main objective of this study is to compare urinary electrophoretic patterns among a healthy population (G_0_), dogs with azotemia and proteinuria due to CKD not related to *L. infantum* infection (G_1_), and dogs with CKD related to *L. infantum* infection (G_2_) and the evaluation of UCE as a promising laboratory technique for the study and monitoring of proteinuria in dogs.

## Materials and methods

### Study population and sample collection

The study population was located in the Mediterranean area of Spain including outdoor and indoor dogs, 123 urine samples from seronegative healthy dogs were included as a control group (G_0_). Healthy dogs had to meet the following criteria: anamnesis without abnormalities, normal physical examination, and within reference limits laboratory results. Twenty-eight urine samples from dogs with azotemia and proteinuria were collected between December 2017 and April 2019. Eleven samples of dogs with CKD not related to *L. infantum* (G_1_) were collected. Dogs were classified according to the IRIS guidelines, CKD was staged based on blood creatinine, and substaged by proteinuria (21). Seventeen samples from dogs with CKD related to *L. infantum* infection (G_2_) were collected and classified according to the LeishVet clinical staging (22), subdividing individuals into Stage I (mild disease: dogs with mild clinical signs, such as solitary lymphadenomegaly or papular dermatitis, and antibody levels from negative to low positive); Stage II (moderate disease: dogs, which apart from the signs listed in Stage I, may have diffuse or symmetrical cutaneous lesions, such as exfoliative dermatitis/onychogryphosis, ulcerations, generalized lymphadenomegaly, loss of appetite, weight loss, and antibody levels from low to high positive); Stage III (severe disease: dogs, which apart from the signs listed in Stages I and II, may present signs originating from immune complex lesions and antibody levels from medium to high positive); and Stage IV (very severe disease: dogs with clinical signs listed in Stage III, pulmonary thromboembolism, nephrotic syndrome, or end-stage renal disease, and antibody levels from medium to high positive). The study was approved by the research and Ethics Committee of the Universidad Católica de Valencia San Vicente Mártir (Valencia, Spain; UCV 2017–2018–33). Inclusion criteria, such as age, sex, breed, reproductive status, or preventive care were randomly included and not relevant to the study. Complete blood count, serum biochemistry, serum capillary electrophoresis, complete urinalysis, and immunofluorescence antibody test (IFAT) serology to detect antibody titers for *L. infantum* were performed. Dogs with concurrent diseases (urinary tract infection, pyelonephritis, severe liver disease, heart disease, or cancer) or medications (diuretics, systemic steroids, angiotensin converting enzyme inhibitors, or nonsteroidal anti-inflammatory) at the time or 15 days before that could interfere with laboratory results were excluded.

Blood samples were collected from either the cephalic or jugular vein after 12 h of fasting. A minimum of 2 ml of blood was collected from each dog. To preserve the sample until analysis, a 0.5 ml EDTA tube (Aquisel, Barcelona, Spain) and a 2 ml tiger-top tube (Aquisel, Barcelona, Spain) were used. All samples were analyzed within the following 24 h by a reference laboratory (Cedivet, Valencia, Spain). All urine samples were collected by free catch in a 150 ml sterile plastic cup (Deltalab, Barcelona, and Spain). Most samples were collected by owners, and fewer samples were collected by the veterinarian if the dog was hospitalized at the time of sample collection. Eight milliliter was stored at 4°C until urinalysis was performed within the next 12 h.

The analysis included a complete blood count (Celltac Alpha VET MEK-6550; Nihon Kohden Ibérica, Madrid, Spain) with a blood smear evaluation. Biochemical analytes measured included creatinine, urea, alanine aminotransferase, and total serum proteins (CS 300 analyzer; Dirui, Jilin, P.R., China). Serum phoretograms were run by capillary electrophoresis (Minicap instrument; Sebia, Barcelona, Spain). Sera of all animals was also tested for antibodies to *L. infantum*, by the indirect fluorescence antibodies test (IFAT) (22) and examined in a fluorescence microscope at 540 nm wavelength (Axio Scope HBO 50 microscope; Zeiss, Madrid, Spain).

Urine samples were centrifuged (Centrifuge 2,650; Nahita, La Rioja, Spain) at 804 × *g* for 5 min, and the supernatants were divided into aliquots of 4 mL and stored at −20°C prior to dialysis in a 1.5 ml plastic microcentrifuge tube (Lambda, Paterna, Spain). The remaining volume of urine was used to evaluate the following variables: microscopic fresh and stained sediment at low- and high-power fields (Binocular microscope DM500; Leica), urine total proteins, and creatinine (automated chemistry analyzer; Dirui, Jilin, P.R., China), to allow the calculation of the urinary protein to creatinine ratio (UPC) (urine creatinine dilution was 1:49 in all samples, creatinine was measured by a creatinine enzymatic method, and proteins in urine were measured with pyrogallol red reagent), urine culture in a specific chromogenic medium (CHROMagar orientation medium; Becton Dickinson, Madrid, Spain), specific gravity (refractometer; Optika Ponteranica, Ponteranica BG, Italy), and pH, glucose, ketone, bilirubin, and hemoglobin levels (LabStrip U11 Plus; 77 Elektronika, Budapest, Hungria). Animals that presented active sediments and samples exceeding 100,000 colony-forming units (CFUs) were excluded from the study.

### Urinary capillary electrophoresis (UCE)

Urinary capillary electrophoresis (UCE) was performed according to the standardized method described by Navarro et al. in 2021. Urine was dialyzed before CE (Sebia, Barcelona, Spain) to eliminate compounds that could interfere with the wavelength used for reading and cause artifact peaks. Additionally, the dialization process eliminates the presence of salts that could stick to the capillary tube. The dialysis procedure was performed following the manufacturer's protocol. Urine supernatant (4 ml) from each dog was thawed at room temperature and centrifuged at 1,609 × *g* for 10 min. The resulting supernatant was transferred to a 4 ml dialysate column (Vivaspin Turbo 4 10,000 MWCO; Sartorius, Goettingen, Germany). These columns have a collection tube that includes a filter that removes unwanted compounds and concentrates the number of proteins in the sample. Dialysate columns containing urine were centrifuged at 1,878 × *g* for 25 min, and the urine that passed through the filter to the container was discarded. A washing solution was prepared by adding 50% distilled water and 50% dialysis buffer (Sebia, Barcelona, Spain) in a sterile container. The concentrator was refilled up to 4 ml volume with this solution and centrifuged at 1,878 × *g* for 20 min. The remaining 200 μl solution in the collection tube was transferred to a 1.5 ml microcentrifuge tube and subjected to CE (Minicap; Sebia, Barcelona, Spain).

**Figure 1:**
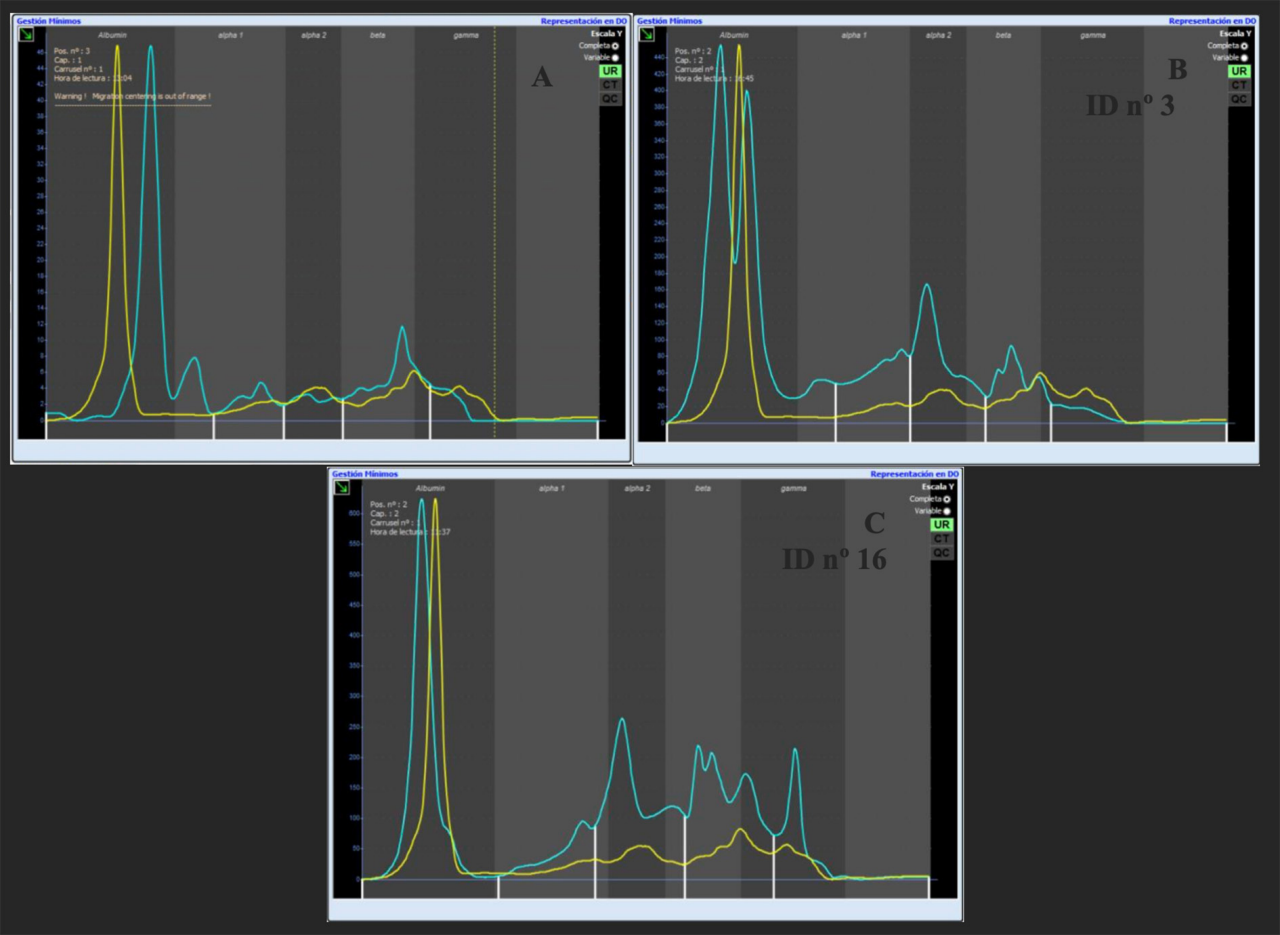
**(A)** Overlay of canine serum diluted 1:49 with the urinary phoretogram of a healthy dog; **(B)** Overlay of canine serum diluted 1:49 with the urinary phoretogram of a dog with CKD (G_1_); **(C)** Overlay of canine serum diluted 1:49 with the urinary phoretogram of a dog with CKD associate to *L. infantum* infection (G_2_). The yellow line represents the canine diluted serum and the blue line represents the de electrophoretic pattern of the studied urine.

As electrophoresis quality control, frozen aliquoted sera from a healthy dog were included, diluted in running buffer at 1:49, and migrated prior to any run and in each batch (16). The graph obtained from the electrophoresis of a non-pathological diluted canine serum (curve pattern) was placed over all urinary electrophoretic graphs of healthy dogs and dogs with CKD (Figure 1). This diluted serum curve provided a guide to separate the different urinary fractions in the total pathological population.

The urine electrophoretic pattern obtained for each dog was divided into protein fractions 1 to 5 (F1–F5). These fractions were determined by superimposing the normal canine diluted serum over the electrophoretic samples, this way, F1 corresponded with albumin, F2 corresponded with alpha_1_-globulin, F3 corresponded with alpha_2_-globulin, F4 corresponded with beta-globulin, and F5 corresponded with gamma globulin. Protein fractions were verified and, if necessary, corrected by visual inspection of the electrophoretogram. All samples were analyzed by the same person (Supplementary material 1).

### Statistical evaluation

Statistical analysis was performed using the R commander program (R v.3.4.3; https://www.r-project.org/). The Anderson–Darling test was used to test the hypothesis of normality. In this study, the results were considered statistically significant if *p* < 0.05. Outliers were detected by boxplot and eliminated if they were considered aberrant observations, although the emphasis was to retain them rather than delete them. The one-way ANOVA test was used to establish differences between groups. Linear regression was performed to predict the value of urinary fractions of the phoretogram based on the value of serum fractions, as well as between urinary fractions of the phoretogram and the UPC.

## Results

### Study population

Control group clinical findings (G_0_)

G_0_ included 123 samples from healthy dogs, 54 males, and 69 females and the range of age was 5 months to 13 years, and the median was 6.5. No significant hematologic or biochemical abnormalities were found in this population. Regarding serum protein electrophoresis, values within the reference range were observed for the different fractions, except in dogs older than 7 years. In 16.26% of these animals, a polyclonal increase in the gamma globulin fraction (RI: 6–12%) was found. All dogs tested negative for *L. infantum* antigen.

#### Dogs with CKD not related to *L. Infantum* (G_1_)

G_1_ included 11 samples, 4 males, and 7 females, and the range of age was 4 years to 13 years, and the median was 9. Six dogs (54.54%) presented mild to moderate normocytic normochromic non-regenerative anemia. Dogs were classified according to the IRIS classification: 4/11 (36.36%) IRIS II, 2/11 (18.18%) IRIS III, and 5/11 (45.45%) IRIS IV. All dogs tested negative for *L. infantum* antigen. In serum phoretogram, 3/11 dogs (27%) showed hypoalbuminemia and increases of 4/11 (36.36%) in the alpha_1_-globulin fraction, 4/11 (36.36%) in the alpha_2_-globulin fraction, 6/11 (54.55%) in the beta globulin fraction, and 7/11 (63.64%) in the gamma globulin fraction. Urine was isosthenuric (urine specific gravity (USG) 1.008–1.012) in 7/11 dogs (63.63%) and in 4/11 (27.27%) was moderately concentrated (USG 1.013–1.029). The UPC mean value in this group was 1.87 classifying all dogs as proteinuric (Table 1).

**Table 1:** Main analytical results of hematology, biochemistry, and urinalysis of the dogs included in the group with CKD (G_1_).

**Individual**	**PCV (37-55%)**	**WBC (5.5-17.0 K/μL)**	**PTL (150-500 K/μL)**	**Creatinine (0.5-1.60 mg/dL)**	**Urea (18–60 mg/dL)**	**UPC**	**USG**
1	28.4	9.2	331	3.50	243	1.68	1.012
2	21.6	5.78	127	10.82	619	1.5	1.010
3	39.4	4.85	389	2.71	150	2.61	1.018
4	28.2	11.4	338	9.60	379	4.67	1.014
5	42.6	13.8	202	7.74	409	3.22	1.012
6	29.0	17.0	380	5.41	257.1	0.7	1.008
7	43.4	20.3	1,088	4.48	265	3.63	1.012
8	37.0	8.9	430	1.74	57	0.65	1.020
9	28.4	95	519	3.90	360	0.92	1.012
10	49.5	11.3	626	1.94	156	0.51	1.012
11	34,6	6,9	428	6.14	184	0.52	1.013

(PCV) packed cell volume; (WBC) white blood cell; (PTL) Platelet count; (UPC) protein to creatinine ratio; (USG) urine specific gravity.

#### Dogs with CKD related to *L. infantum* infection (G_2_)

G_2_ included 17 samples, 9 males and 8 females, and the range of age was 10 months to 11 years, and the median was 6.5. Six dogs (35.29%) showed mild normocytic normochromic non-regenerative anemia. All dogs tested positive for *L. infantum* antigen (>1/100). Dogs were classified according to LeishVet clinical staging (22): Stage III severe disease 4/17 (23.52%) and Stage IV very severe disease 13/17 (76.47%). Serum electrophoresis showed 15/17 (82.35%) dogs with decreased albumin fraction and increases in 2/17 (11.77%) in the alpha_1_-globulin fraction, 3/17 (17.65%) in the alpha*2*-globulin fraction, 12/17 (70.59%) in the beta-globulin fraction and 17/17 (100.00%) in the gamma-globulin fraction. Urine specific gravity revealed moderately concentrated urine (USG 1.013–1.029) in 11/17 (64.70%) samples, 5/17 (29.41%) samples were isosthenuric (USG 1.008–1.012) and only one dog (5.88%) had concentrated urine (USG > 1030). The urinary protein to creatinine **(**UPC) mean value in this group was 3.15 classifying all dogs as proteinuric (Table 2).

**Table 2:** Main analytical results of hematology, biochemistry, and urinalysis of the dogs included in the group of CKD associated with *L. infantum* infection (G_2_).

**Individual**	**PCV (37-55%)**	**WBC (5.5-17.0 K/μL)**	**PTL (150-500 K/μL)**	**Creatinine (0.5-1.60 mg/dL)**	**Urea (18–60 mg/dL)**	**UPC**	**USG**
1	35.8	9.3	358	1.8	153	0.5	1.018
2	23.8	5.6	509	2.80	132	9.10	1.020
3	33.6	11.1	186	4.61	228	4.84	1.014
4	24.5	6.9	185	4.40	315	1.2	1.012
5	42.6	8.9	216	2.10	149	3.11	1.012
6	41.9	7.7	213	6.9	497	1.43	1.014
7	27.3	13.7	137	2.10	87	1.5	1.020
8	30.7	11.4	25	1.70	137	1.86	1.044
9	27.0	8.4	184	2.64	112	1	1.010
10	18.3	9.9	418	4.99	296	2.06	1.015
11	32.0	28.4	224	2.84	149	0.77	1.010
12	24.0	24.8	781	1.62	102	0.9	1.020
13	27.6	3.2	29	6.8	245	0.5	1.014
14	36.9	8.7	216	6.81	301	7.81	1.018
15	31,1	13,3	136	4.22	278	1,89	1.028
16	35.8	9.3	358	1.8	153	2.75	1.018
17	23.8	5.6	509	2.80	132	10.01	1.010

(PCV) packed cell volume; (WBC) white blood cell; (PTL) Platelet count; (UPC) protein to creatinine ratio; (USG) urine specific gravity.

### Comparison between healthy dogs (G_0_) and dogs with CKD (G_1_ and G_2_)

**Figure 2:**
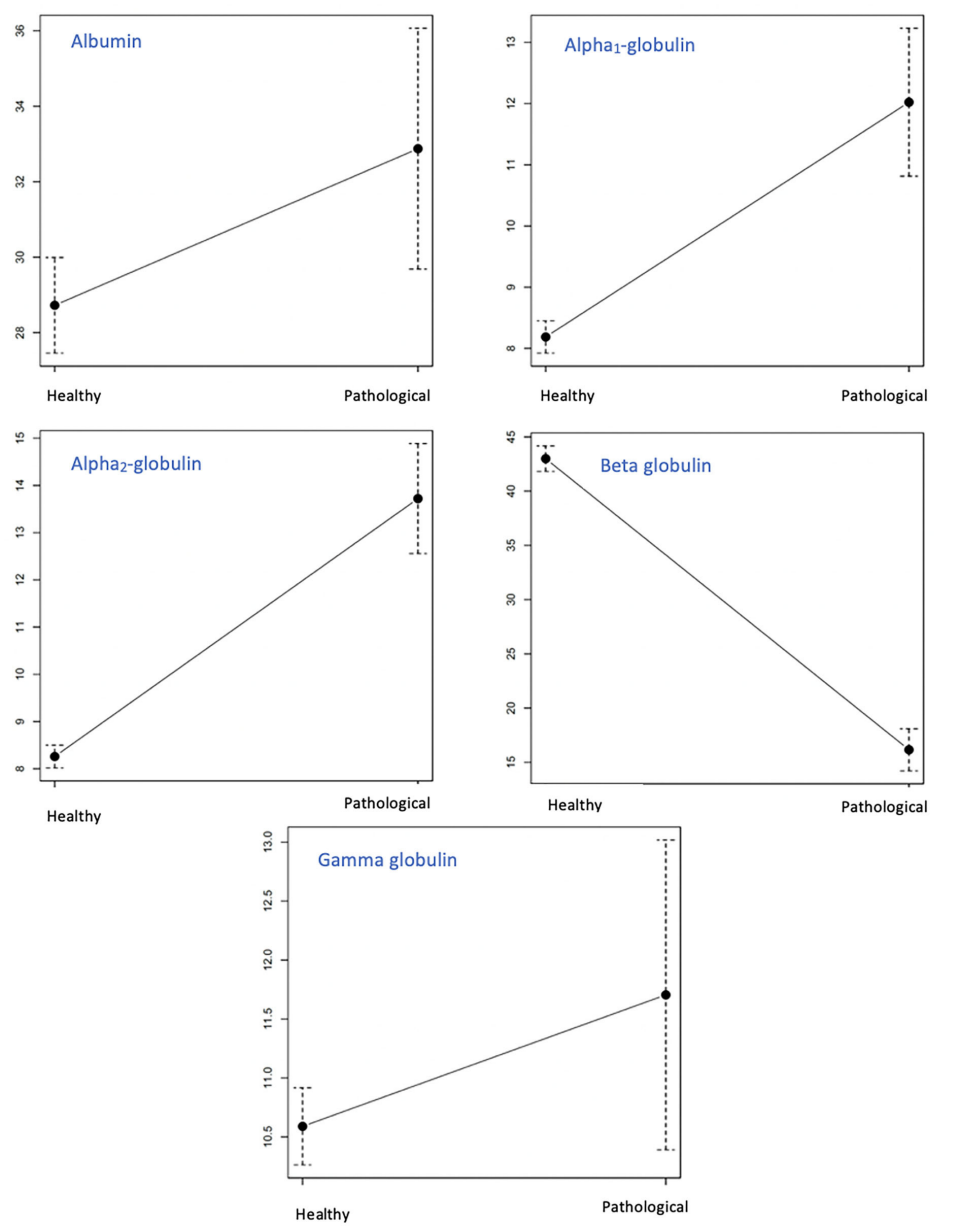
Comparison of the mean values in percentage of all the fractions identified in the urinary phoretogram between dogs with CKD (G_1_ + G_2_) and healthy dogs (G_0_). The Y axis represents the different minimum and maximum values in the percentage of the different populations. The dashed line represents the maximum and the minimum of each group.

A one-way ANOVA comparison was performed between G_0_ (*n* = 123) and G_1_ and G_2_ total population (*n* = 28) to test for possible significant differences between fractions. F2 and F3 were significantly increased in G_1_ and G_2_ compared with F2 and F3 in G_0_. F4 was found to be significantly decreased in G_1_ and G_2_ compared with F4 in G_0_ (Table 3). No significant differences were detected between groups (G_0_ vs. G_1_ and G_2_) in the F1 and F5 fractions (Figure 2).

**Table 3:** Comparison by one-way ANOVA between the different fractions of the urinary phoretogram in healthy dogs (G_0_) and dogs with CKD (G_1_ + G_2_).

**Fraction**	**N**	**Factor**	**Mean**	**SD**	*p*-value^*^
F2	121	G_0_	8.18	2.9	0.004
	26	G_1_ + G_2_	12.02	6.16	
F3	121	G_0_	8.25	2.65	8E-05
	26	G_1_ + G_2_	13.72	6.17	
F4	123	G_0_	42.99	13.01	2E-16
	25	G_1_ + G_2_	16.16	9.68	

F2 fraction 2 corresponding to alpha1-globulins; F3 fraction 3 corresponding to alpha2-globulins; F4 fraction 4 corresponding to beta-globulins; *n* = 151 dogs (123 healthy, 28 CKD except when abnormal values were excluded); ANOVA analysis of variance, SD standard deviation.

* Significance *p*-value < 0.05.

### Comparison among healthy dogs (G_0_), dogs with CKD not related to *L. infantum* infection (G_1_), and dogs with CKD due to *L. infantum* infection (G_2_)

**Figure 3:**
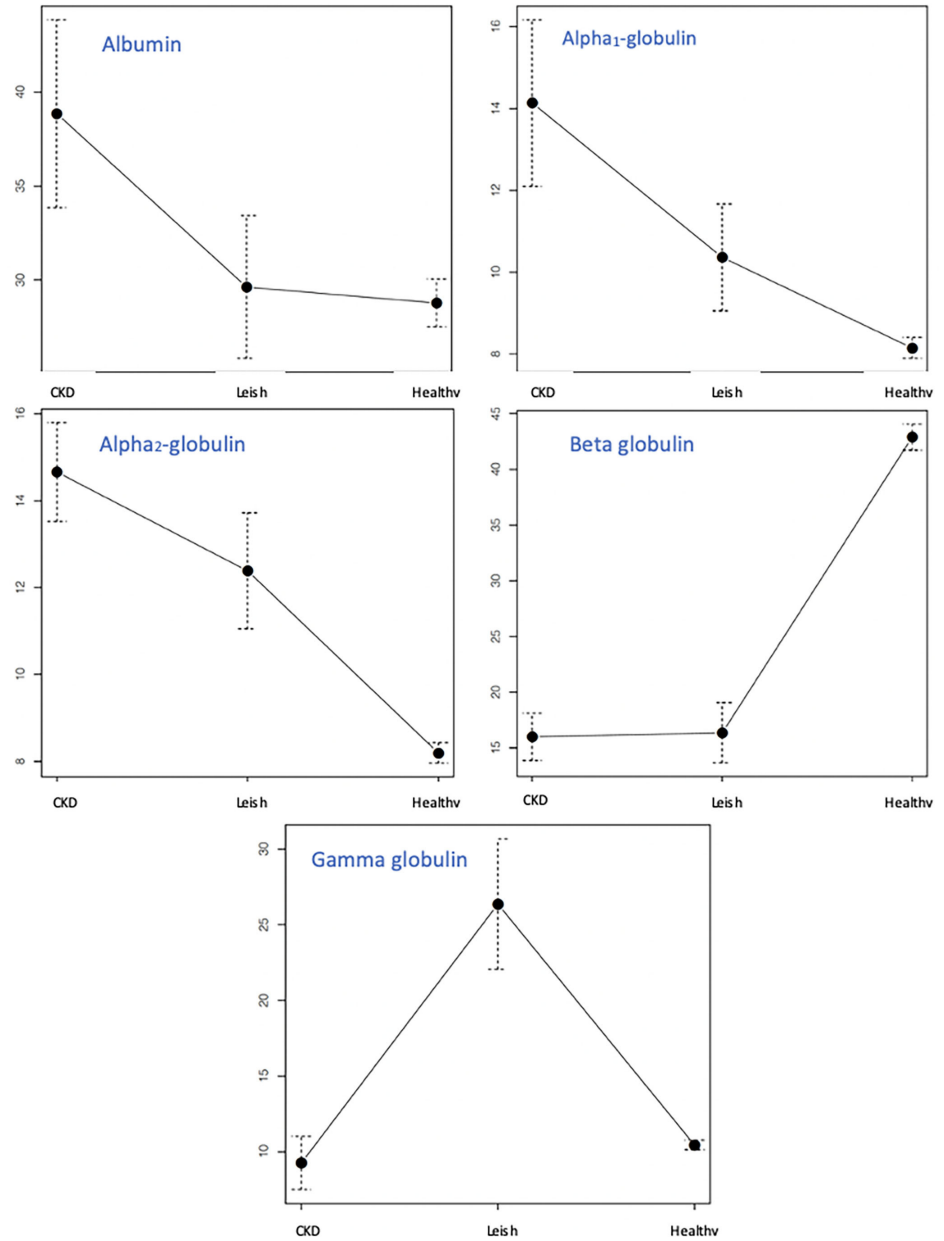
Comparison of the mean values in the percentage of all the fractions identified in the urinary phoretogram among healthy dogs (G_0_), dogs with CKD (G_1_), and dogs with CKD associated with *L. infantum* infection (G_2_). The Y axis represents the different minimum and maximum values in the percentage of the different populations. The dashed line represents the maximum and the minimum of each group.

A one-way ANOVA comparison was performed among G_0_ (*n* = 123), G_1_ (*n* = 11), and G_2_ (*n* = 17). F2 was significantly higher in G_1_ compared with groups G_0_ and G_2_. F3 was found to be significantly higher in G_1_ and G_2_ compared with G_0_. F4 was significantly lower in G_1_ and G_2_ compared with G_0_, and F5 was significantly higher in dogs with CKD associated with infection by *L. infantum*, G_2_ compared with G_0_ and G_1_ (Table 4). No significant differences were detected for the F1 fraction (Figure 3).

**Table 4:** Comparison by one-way ANOVA between the different fractions of the urinary phoretogram in healthy dogs (G_0_), dogs with CKD not related to *L. infantum* infection (G_1_), and dogs with CKD associated with *L. infantum* infection (G_2_).

**Fraction**	***N***	**Factor**	**Mean**	**SD**	*p*-value^*^
F2	121	G_0_	8.18	2.9	0.002
	11	G_1_	15.53	2.94	
	14	G_2_	9.6	6.05	
F3	121	G_0_	8.25	2.65	6E-04
	11	G_1_	13.25	3.5	
	17	G_2_	12.44	5.34	
F4	123	G_0_	42.99	13.01	1E-14
	11	G_1_	23.92	13.56	
	17	G_2_	22.95	13.86	
F5	116	G_0_	10.5	3.43	0.005
	11	G_1_	7.8	5.74	
	17	G_2_	22.48	13.77	

F2 fraction 2 corresponding to alpha1-globulins; F3 fraction 3 corresponding to alpha2-globulins; F4 fraction 4 corresponding to beta-globulins; F5 fraction 5 corresponding to gamma globulins; *n* = 151 dogs (123 healthy, 11 with chronic kidney disease, 17 *L. infantum* except when abnormal values were excluded); ANOVA analysis of variance, SD standard deviation.

* Significance of *p*-value < 0.05.

### Relationship between UPC and the different fractions in G_1_ and G_2_

Linear regression was performed to predict the value of urinary fractions of the phoretogram based on the value of UPC. No relation was found between the different fractions of the urinary phoretogram and the UPC of each dog belonging to G_1_ and G_2_ (Table 5).

**Table 5:** Linear regression performed to predict the relationship between different fractions of the urinary phoretogram and UPC in dogs with CKD not related to *L. infantum* infection (G_1_).

**Fraction G_1_**	*p*-value^*^	**UPC Mean**	**UPC SD**	**Fraction G_2_**	*p*-value^*^	**UPC Mean**	**UPC SD**
F1	0.972	1.87	1.44	F1	0.641	3.15	2.99
F2	0.919			F2	0.072		
F3	0.660			F3	0.283		
F4	0.914			F4	0.228		
F5	0.609			F5	0.269		

F1 fraction 1 corresponding to albumin; F2 fraction 2 corresponding to alpha1-globulins; F3 fraction 3 corresponding to alpha2-globulins; F4 fraction 4 corresponding to beta-globulins; F5 fraction 5 corresponding to gamma globulins.

* Significance of *p*-value < 0.05.

Linear regression performed to predict the relationship between different fractions of the urinary phoretogram and UPC and dogs with CKD associated with *L. infantum* infection (G_2_).

### Relationship between serum fractions and urine fractions in G_1_ and G_2_

Linear regression was performed to predict the value of urinary fractions of the phoretogram based on the value of serum fractions. No relation was found between the different fractions of the serum and urinary phoretogram of each dog belonging to G_1_ and G_2_ (Table 6).

**Table 6:** Linear regression performed to predict the relationship between different fractions of the serum and urine phoretogram in dogs with CKD not related to *L. infantum* infection (G_1_).

**Fraction G_1_**	*p*-value^*^	**Urine Mean**	**Urine SD**	**Fraction G_2_**	*p*-value^*^	**Urine Mean**	**Urine SD**
F1	0.565	38.85	16.56	F1	0.618	29.00	16.41
F2	0.576	14.13	6.754	F2	0.715	13.22	9.261
F3	0.407	16.24	6.269	F3	0.366	12.08	5.698
F4	0.310	21.50	13.80	F4	0.646	18.77	15.02
F5	0.209	9.254	5.842	F5	0.055	26.90	18.70

F1 fraction 1 corresponding to albumin; F2 fraction 2 corresponding to alpha1-globulins; F3 fraction 3 corresponding to alpha2-globulins; F4 fraction 4 corresponding to beta-globulins; F5 fraction 5 corresponding to gamma globulins.

* Significance of *p*-value < 0.05.

Linear regression performed to predict the relationship between different fractions of the serum and urine phoretogram in dogs with CKD associated with *L. infantum* infection (G_2_).

## Discussion

Urinary capillary electrophoresis (UCE) is an available laboratory technique that can provide an easily interpretable electrophoretic pattern divided into five fractions as in serum (fraction 1 corresponding to the albumin migration zone, fraction 2 corresponding to the alpha1-globulins zone, fraction 3 corresponding to the alpha2-globulins zone, fraction 4 corresponding to beta-globulins zone, fraction 5 corresponding to the gamma globulins zone) which can be useful in the early diagnosis of the renal disease (16, 20).

Regarding serum electrophoresis results, the polyclonal increase observed in the gamma globulin fraction in 16.26% of the control dogs older than 7 years, could be associated with oral diseases and/or chronic joint degenerative pathologies that were recorded during their clinical examination, conditions that are common in animals of that age (23, 24).

Although the origin of the kidney disease in this study was different, a comparison was made with the dogs with CKD (G_1_ and G_2_) to have a larger sample size. Considering all the dogs with CKD (G_1_ and G_2)_, F2 migrating in the zone of alpha_1_-globulins appears to be significantly increased in dogs with kidney disease. In agreement with the present results of this study, Pelander et al. (20) and Gonzalez et al. (25) have reported elevations of certain proteins in CKD patients with or without *L. infantum* infection using capillary electrophoresis coupled to mass spectrometry (CE-MS). One of these proteins is retinol binding protein (belonging to the alpha-1 globulin group of proteins), which is related to proximal tubular dysfunction (26).

When G_1_ and G_2_ groups were compared independently with the reference population, F2 was significantly higher in dogs with CKD non-related to *L. infantum* infection (G_1_). This could be due to the presence of tubular damage in this pathology (2). This hypothesis could be supported since most of the dogs in this group (63.6%) had UPC values lower than 2, which may be more related to tubular proteinuria associated with low molecular weight proteins (< 65 kDa) (2, 9). However, it would be necessary to evaluate more dogs with proteinuria to confirm this hypothesis.

In the present study, F3 (alpha_2_-globulin migrating zone) was significantly increased in urine from *L. infantum* infected and noninfected dogs suffering CKD compared to the control group. Gonzalez et al. also reported that haptoglobin and leucine-rich alpha-2-glycoprotein 1 (LRG1) are increased in these sick dogs (25). Haptoglobin is an acute phase protein, and it is increased in the serum of dogs with leishmaniosis (27). In urine, it has been related to acute kidney disease in babesiosis (28). LRG1 is an acute phase plasma protein related to tubular damage (29). In human medicine, an isoform of retinol binding protein 4 (RBP4-LL) can also be found in F3, and its excretion in urine is also associated with tubular damage. Furthermore, in humans, alpha2-macroglobulin has been found in the urine of patients with severe proteinuria, which could be indicative of glomerular proteinuria (30).

F4 migrating in the zone of beta-globulins was decreased in *L. infantum* infected and non-infected dogs suffering CKD compared with G_0_. It has been recently described that there are three proteins that decrease in the urine of dogs infected with *L. infantum* and suffering from CKD (25). These proteins are Tamm–Horsfall protein (uromodulin), junctional adhesion molecule 1, and myelin protein zero like 1. Studies in human medicine about renal biomarkers show that the Tamm–Horsfall protein may be in this protein fraction. In healthy humans and dogs, uromodulin excretion is normal, and since it has defensive properties against urinary tract infections, there is a negative correlation between its detection and the presence of kidney disease in advanced stages (1, 31, 32). More research is needed to verify whether these proteins migrate into the beta-globulin zone in the CE of canine urine. Beta_2_-microglobulin, which is a renal biomarker for the early stages of kidney disease, can also be found in the F4 migration zone (33). However, in this study, dogs with CKD disease did not follow a homogeneous distribution in terms of IRIS classification, and dogs with stage I were not included, which made it difficult to assess beta_2_-microglobulin involvement.

F5 was significantly higher in dogs with CKD associated with *L. infantum* infection. There are several studies that confirm the presence of immunoglobulin G in dogs' urine infected with *L. infantum* and glomerulonephritis (6, 34). This is one of the reasons why UCE in dogs infected by *L. infantum* could be useful in the diagnosis and the follow-up of its medical treatment. More studies will be necessary to evaluate the variation of this fraction depending on the different stages of canine leishmaniosis and its evolution after the administration of the different treatment options.

In this study, F1 was not statistically significant when comparing G_0_, G_1_, and G_2_, although there are several studies that describe dogs with kidney disease, albumin is excreted in greater quantity (2–4, 32). This could be due to a beta error because of the small size of the study population, and a more homogenic group regarding the IRIS stage would be necessary.

Comparison between different urinary fractions of the dog's phoretogram and its UPC did not show statistically significant results. Although a positive correlation between a higher UPC and one or more elevated fractions seems logical, this association was not observed. In the authors' opinion, these results could be explained by the fact that in G_1_, 63.6% of the dogs had UPC < 2 and in G_2_, 53% had UPC < 2, being low values for this parameter in animals with CKD or leishmaniosis. It would be interesting to evaluate a larger study population with different UPC values.

Comparison between the different serum and urine fractions of the same dog did not show any significance, this could be because in this study lower serum albumin values did not necessarily correspond to dogs with higher UPC. In dogs with CKD due to *L. infantum* infection, 100% had higher values in serum gamma globulins and did not correlate with a higher urinary excretion of this fraction. This could be explained, as in the other fractions, because higher serum values are not necessarily related to greater protein excretion through urine. More studies would be necessary for which capillary electrophoresis associated with mass spectrophotometry would be used to evaluate the presence of proteins in each fraction and assess whether the proteins are the same in the different fractions of the urine and serum phoretograms. In the study carried out by Pelander et al. in 2019, it was detected that most differentially excreted peptides represented fragments of collagen I, indicating a possible association with fibrotic processes in CKD (similar to the equivalent human urinary peptide CKD model). Another alternative to evaluate which proteins are present in each fraction of the urinary phoretogram is the characterization of proteins by electrophoresis and immunofixation, which can be a useful complement to standard biochemical techniques for the characterization of serum and urinary proteins (35).

A limitation of the study is the impossibility to compare these CE results with other studies that use other urine electrophoresis techniques since they follow different principles. It would be interesting to associate the UCE technique with mass spectrophotometry or immunofixation, to assess which proteins are excreted in each fraction of the urinary phoretogram. These proteins could be used as a urinary biomarker for the diagnosis and monitoring of different pathologies. If we would be capable of correlating the major proteins with their corresponding CE urinary fraction, CE could be used routinely in diagnostic laboratories for prognostic and monitoring purposes, as well as point to a tubular, selective, or non-selective glomerular or mixed proteinuria. Another limitation has been detected in Terms of the study population. Collecting pathological samples has been a challenge, as these dogs did not have to receive any prior treatment that could interfere with blood and urine tests. It would be interesting to enlarge the study population by standardizing the population of dogs with kidney disease, based on the IRIS classification and the LeishVet clinical staging (21, 22). The present study compared quantitative values of the urinary fractions from G_1_ and G_2_ with the reference intervals published previously (16).

## Data availability statement

The raw data supporting the conclusions of this article will be made available by the authors, without undue reservation.

## Ethics statement

The animal study was reviewed and approved by the research and Ethics Committee of the Universidad Católica de Valencia San Vicente Mártir (UCV 2017–2018–33). Written informed consent was obtained from the owners for the participation of their animals in this study.

## Author contributions

LG and SF-B designed the study. PN collected the data, and exported, structured, and checked the data from the laboratory information system. LG, SF-B, and PN wrote the paper, analyzed and interpreted the data, and reviewed the manuscript. All authors read and approved the final manuscript.

## Funding

This study was supported by Universidad Católica de Valencia San Vicente Mártir (grant UCV 2016-226-001/UCV 2018-226-001).

## Conflict of interest

The authors declare that the research was conducted in the absence of any commercial or financial relationships that could be construed as a potential conflict of interest.

## Publisher's note

All claims expressed in this article are solely those of the authors and do not necessarily represent those of their affiliated organizations, or those of the publisher, the editors and the reviewers. Any product that may be evaluated in this article, or claim that may be made by its manufacturer, is not guaranteed or endorsed by the publisher.

## Supplementary material

Supplementary Material 1 Comparison between phoretograms with the five fractions of dogs with CKD **(A)** and dogs with CKD associated with L.infantum **(B)**.
