# Supplementary figures and images for: Urinary phoretograms performed by capillary electrophoresis in dogs with chronic disease with or without Leishmania infantum infection
Source: Front Vet Sci. 2022 Nov 18;9:979669. doi: 10.3389/fvets.2022.979669 (PMC9716102; doi:10.3389/fvets.2022.979669)

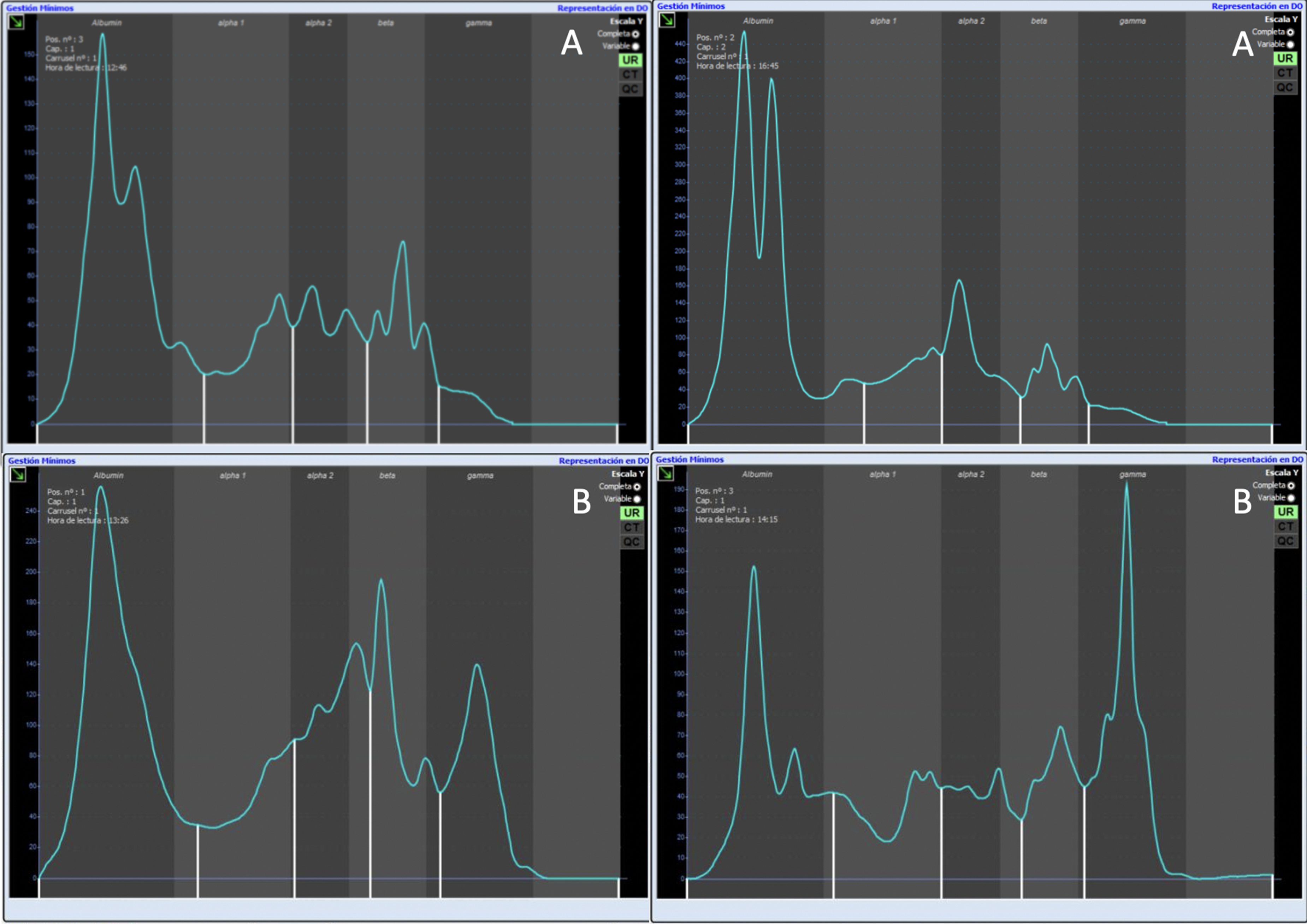

Supplement: Supplementary Material 1 — Comparison between phoretograms with the five fractions of dogs with CKD (A) and dogs with CKD associated with L.infantum (B). [file Image_1.TIFF]
